# Identification of a functional missense variant in the matrix metallopeptidase 10 (*MMP10*) gene in two families with premature myocardial infarction

**DOI:** 10.1038/s41598-024-62878-3

**Published:** 2024-05-28

**Authors:** Viktor Verovenko, Stephanie Tennstedt, Mariana Kleinecke, Thorsten Kessler, Heribert Schunkert, Jeanette Erdmann, Stephan Ensminger, Zouhair Aherrahrou

**Affiliations:** 1 Institute for Cardiogenetics, University of Luebeck, Luebeck, Germany; 2 DZHK (German Research Centre for Cardiovascular Research) Partner Site Hamburg/Luebeck/Kiel, Luebeck, Germany; 3 University Heart Center, Luebeck, Germany; 4 Menzies School of Health Research and Charles Darwin University, Darwin, Northern Territory 0811, Australia; 5 Department of Cardiology, German Heart Centre Munich, Technical University of Munich, Munich, Germany; 6 DZHK (German Center for Cardiovascular Research), Partner Site Munich Heart Alliance, Munich, Germany; 7 Clinic for Cardiac and Thoracic Vascular Surgery, UKSH (University Hospital Schleswig-Holstein), Luebeck, Germany

**Keywords:** Computational models, Disease genetics, Cardiovascular biology, Cardiovascular diseases, Inflammation, Genetics research, Cell adhesion, Cell migration, Gene expression, Mutation

## Abstract

A positive family history is a major independent risk factor for atherosclerosis, and genetic variation is an important aspect of cardiovascular disease research. We identified a heterozygous missense variant p.L245P in the *MMP10* gene in two families with premature myocardial infarction using whole-exome sequencing. The aim of this study was to investigate the consequences of this variant using in-silico and functional in-vitro assays. Molecular dynamics simulations were used to analyze protein interactions, calculate free binding energy, and measure the volume of the substrate-binding cleft of MMP10-TIMP1 models. The p.L245P variant showed an altered protein surface, different intra- and intermolecular interactions of MMP10-TIMP1, a lower total free binding energy between MMP10-TIMP1, and a volume-minimized substrate-binding cleft of MMP10 compared to the wild-type. For the functional assays, human THP-1 cells were transfected with plasmids containing *MMP10* cDNA carrying the p.L245P and wild-type variant and differentiated into macrophages. Macrophage adhesion and migration assays were then conducted, and pro-inflammatory chemokine levels were evaluated. The p.L245P variant led to macrophages that were more adherent, less migratory, and secreted higher levels of the pro-inflammatory chemokines CXCL1 and CXCL8 than wild-type macrophages. Thus, the p.L245P variant in *MMP10* may influence the pathogenesis of atherosclerosis in families with premature myocardial infarction by altering protein - protein interactions, macrophage adhesion and migration, and expression of pro-inflammatory chemokines, which may increase plaque rupture. These results could contribute to the development of selective MMP10 inhibitors and reduce the risk of atherosclerosis in families with a history of premature myocardial infarction.

## Introduction

Coronary artery disease (CAD) and the underlying process of atherosclerosis are the leading causes of death worldwide^1^. CAD is caused by the accumulation of atherosclerotic plaques, leading to acute (ACS) and chronic coronary syndromes according to clinical progression^2^, with ACS including acute myocardial infarction (MI), unstable angina, and sudden cardiac death^3^. Plaque rupture is the most common pathophysiological cause of MI, resulting in unstable plaques and coronary thrombus^4,5^. A positive family history is considered an independent risk factor for genetic susceptibility and the pathogenesis of atherosclerosis^6^. Screening for disease-causing variants in patient studies led to the identification of a heterozygous missense variant NM_002425:exon5:c.T734C:p.L245P in the *MMP10* gene in two families with premature MI before the age of 60.

**Figure 1:**
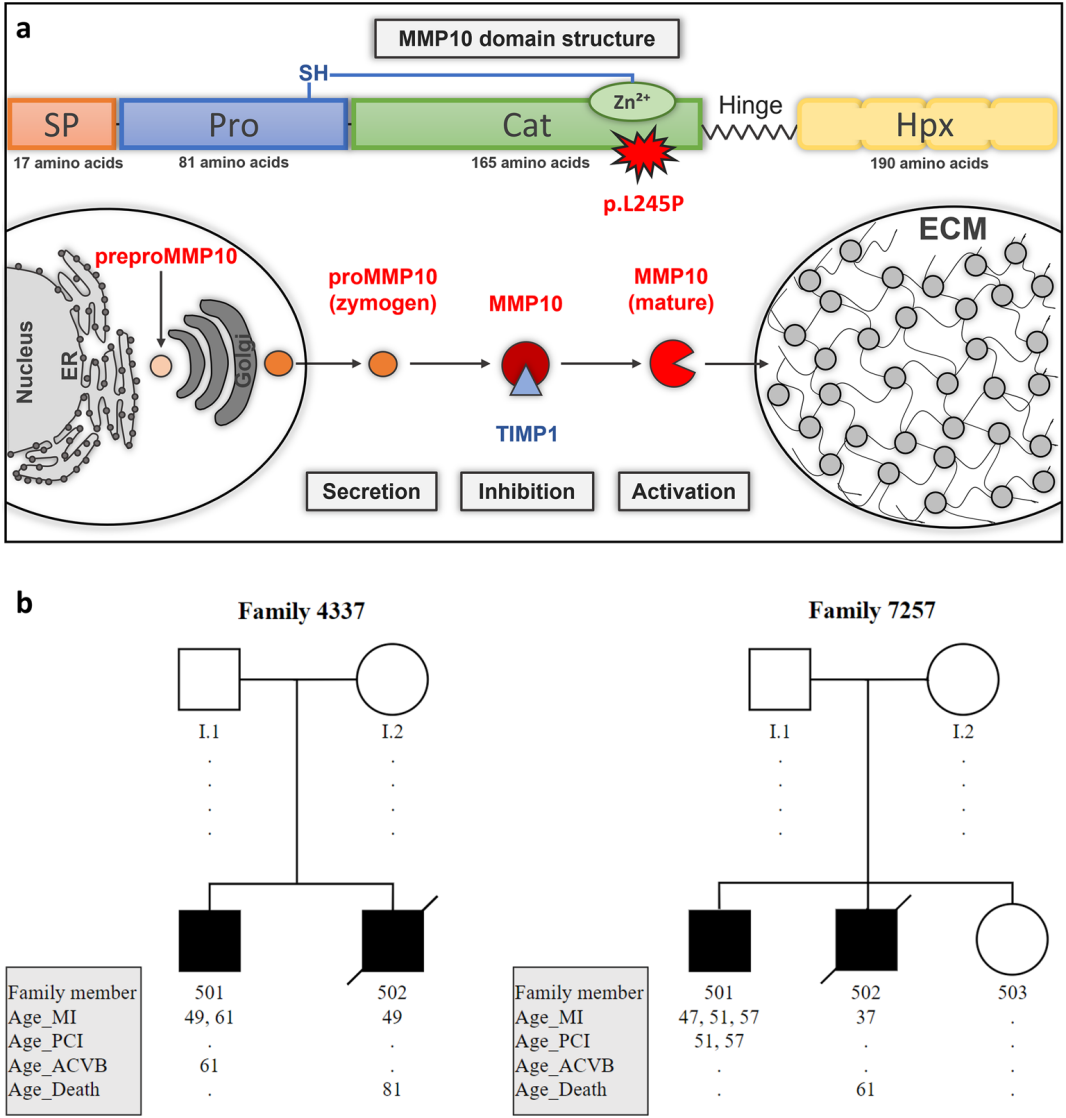
Structure of MMP10 domain and pedigrees of two families with the p.L245P variant. (**a**) Representation of the domain structure of MMP10 with the variant p.L245P, showing SP =  signal peptide; Pro = pro-domain; Cat = catalytic domain; Hpx = haemopexin domain with the respective number of amino acids. Schematic representation of intracellular synthesis, extracellular secretion, and activation/inhibition of mature MMP10 by TIMP1 in equilibrium. (**b**) Representation of two family pedigrees. Legend of symbols: Square = male; circle = female; crossed-out square = member deceased; black = affected; white = healthy; I.1/I.2 = family members did not participate in the study; 501–503 = family members included in the study; MI = myocardial infarction; PCI = percutaneous coronary intervention; ACVB = coronary artery bypass grafting.

Matrix metalloproteinases (MMPs) are proteolytic enzymes that are involved in the degradation of components of the extracellular matrix (ECM) and the modulation of tissue remodeling. Among those, MMP10 is involved in atherogenesis^7^. MMP10 protein expression is first tightly regulated at the transcriptional and protein levels^8^. After synthesis as a pre-proenzyme and secretion into the extracellular space as a proenzyme, protein activation occurs via intermolecular processing (Fig. 1a)^9^. Together with TIMP metallopeptidase inhibitor 1 (TIMP1), MMP10 plays an important role in the degradation of ECM and controls vascular remodeling by affecting cell adhesion, migration, and proliferation^10,11^. Altered protein activity and imbalance in MMP10-TIMP1 homeostasis by gene polymorphism may underlie the pathogenesis of atherosclerosis through increased destruction of ECM, activation of further MMPs and vascular smooth muscle cells (VSMCs), modified macrophage adhesion and migration, modulation of inflammatory responses with increased cytokine and chemokine activity, and thinning of the fibrous cap^12–15^.

Several variants of *MMP* genes leading to loss of protease activity have been implicated in different inherited diseases^16^. A polymorphism in *MMP10* has previously been associated with abdominal aortic aneurysms^7^ and vulnerable carotid plaques^17^. Besides, an independent association between elevated measured MMP10 serum levels and subclinical atherosclerosis in asymptomatic patients with cardiovascular risk factors has been demonstrated^18^. Furthermore, an association between MMP10 and the severity and poor outcome of peripheral arterial disease has been shown^19^. In addition, overexpression of MMP10 has been detected in calcified human aortic valves with increased levels of MMP10 in patients' blood^20^, and a significant reduction in atherosclerotic lesion size, macrophages, and plaque calcification has been observed in Apoe (-/-) Mmp10 (-/-) mice^21^.

**Figure 2:**
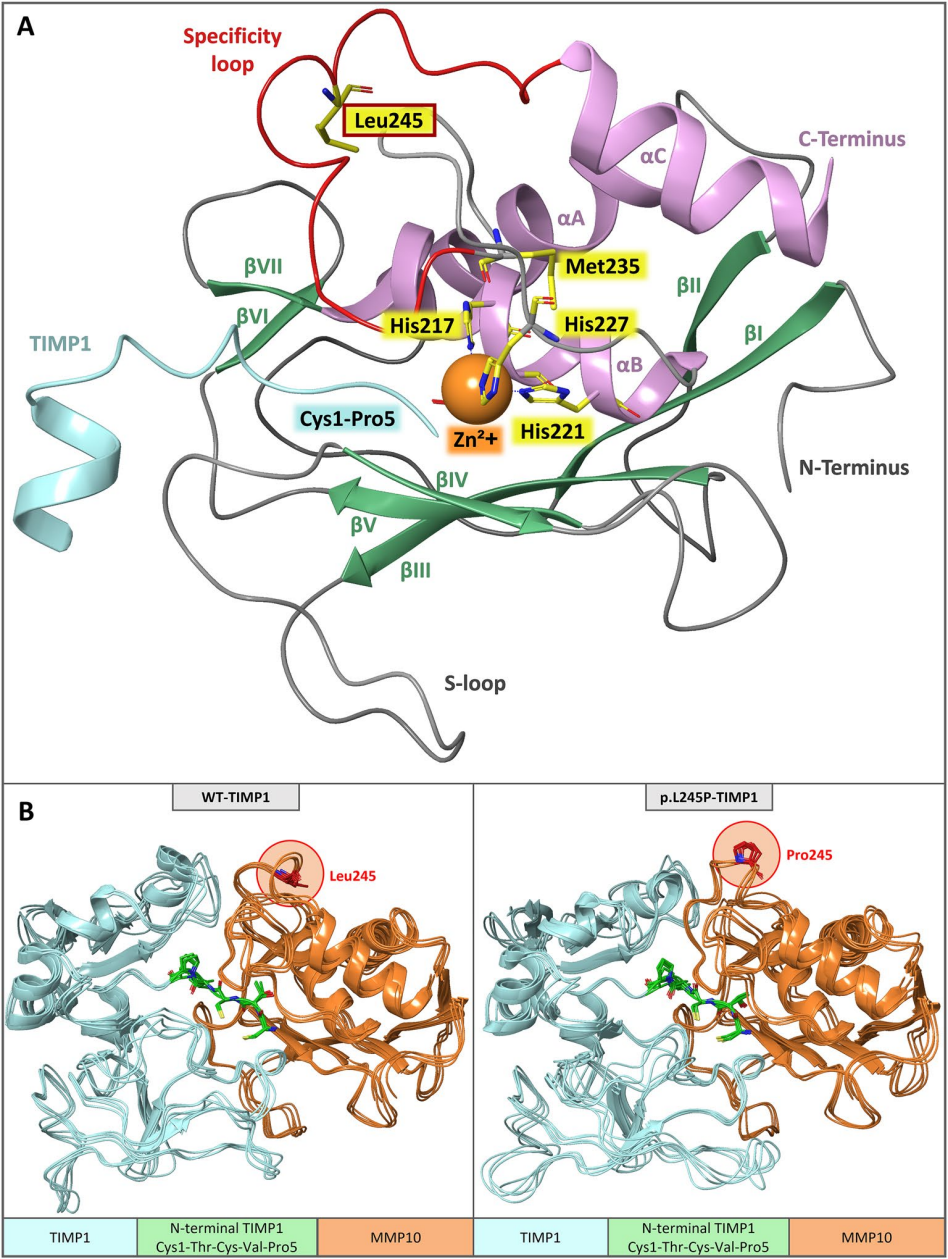
Schematic display of MMP10-TIMP1 and superimposition of snapshots of WT/p.L245P-TIMP1 structures. (**A**) Initial MMP10 WT-TIMP1 structure as ribbon with major segments: Leu245, Met235 of methionine-turn, and histidines as stick (yellow); catalytic zinc ion as ball (orange); TIMP1: N-terminal inhibiting Cys1-Thr-Cys-Val-Pro5 (turquoise); MMP10: β-sheets (green); α-helices (plum); specificity loop (red); s-loop (gray); all other calcium/zinc ions are not shown for a better overview. (**B**) Superimposition of every 10th snapshot of WT/p.L245P-TIMP1 structures (last 200 ns of MD simulation time): TIMP1 (turquoise), TIMP1 N-terminal segment Cys1-Thr-Cys-Val-Pro5 (stick, green), MMP10 (ribbon, orange), and Leu245/Pro245 (stick, red, and circled).

The domain structure of MMP10 consists of a signal peptide, a pro-domain, a catalytic-domain and a hemopexin-domain, each fulfilling a different protein function (Fig. 1a). The p.L245P variant resulted in an amino acid exchange in the region of the specificity loop (Tyr236-Ser251) in the catalytic domain of MMP10 (Figs. 1a, 2). This loop is involved in the formation of the S1-specificity pocket of the substrate-binding cleft of MMP10^22^. A change in the S1-specificity pocket can lead to different substrate specificities of the protein. Therefore, potent and selective MMP inhibitors (MMPIs) based primarily on the S1-specificity pocket have been developed in the past^23^.

This study aimed to elucidate the underlying mechanism by which the p.L245P variant is related to the MI phenotype. Therefore human monocyte-like THP-1 cells were used instead of peripheral human monocytes because they are a well-established cell model with comparatively better reproducibility, easier cell culture, longer lifespan, and more homogeneous behavior^24^. The transfected THP-1 cells were differentiated to study macrophage adhesion, and migration, and to compare the expression of pro-inflammatory chemokines between the identified variant and wild-type (WT) controls. In addition, Molecular dynamics (MD) simulations were performed to investigate the intra- and intermolecular interacting behaviors of MMP10-TIMP1.

## Results

### Identification of p.L245P variant using WES in two families with premature MI

The case subjects in the GerMIFS had an MI before 60 years and at least one first-degree relative with CAD manifestation before 70 years of age^25^. By analyzing the exome data of 255 unrelated patients with MI, we discovered in two unrelated families a variant, NM_002425:exon5:c.T734C:p.L245P, in the *MMP10* gene. This variant was found in four affected individuals but was not prevalent in the unaffected siblings. The affected family members who did not receive a percutaneous coronary intervention (PCI) or coronary artery bypass graft (CABG) after disease onset died at an average age of 71 years, while the affected siblings who received PCI/CABG therapy are currently alive. The children of the affected parents are presently younger than the average age of disease manifestation in their parents (45.5 years) and have not suffered an MI to date (Fig. 1b).

### Molecular dynamics simulations

**Figure 3:**
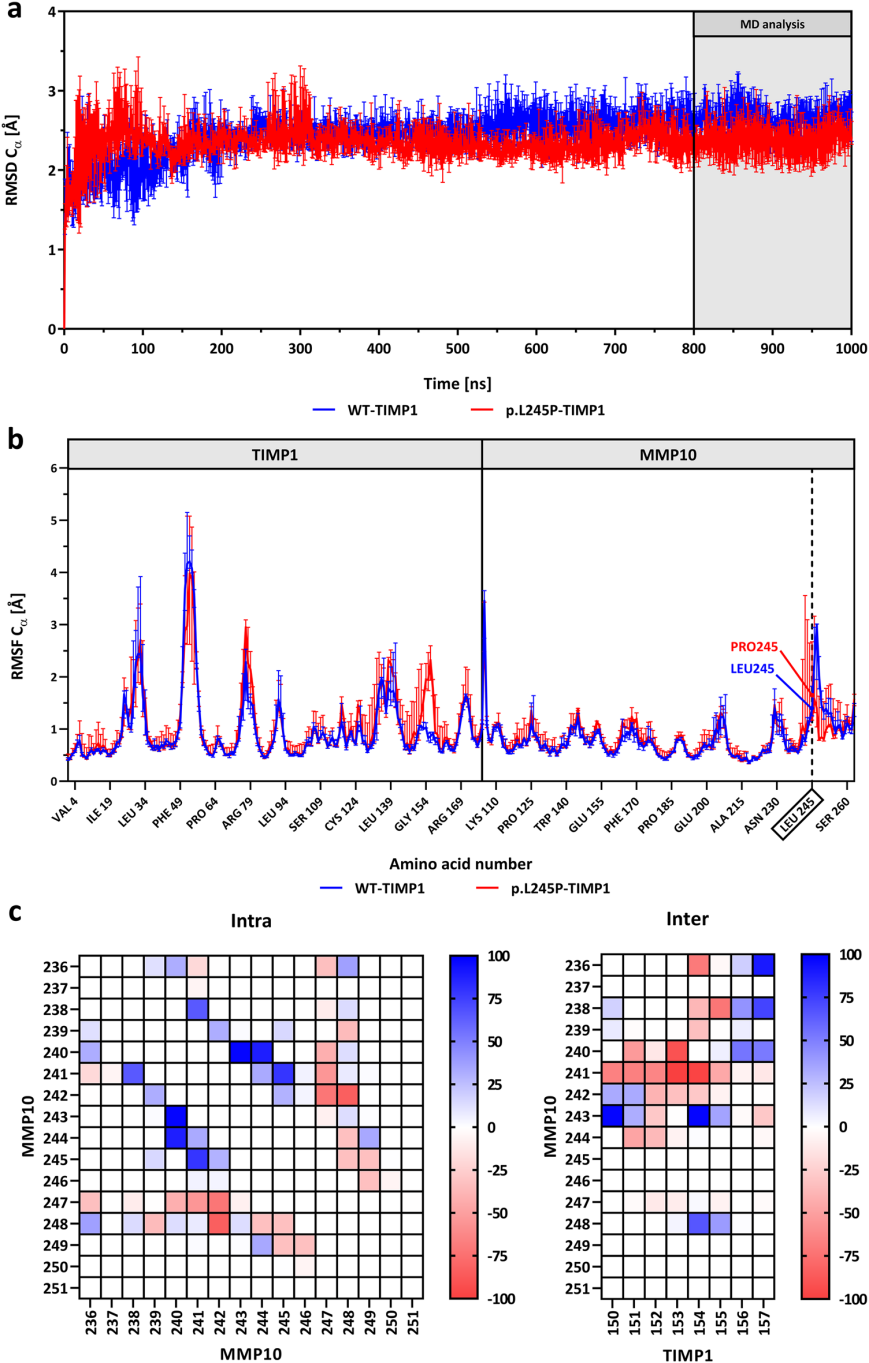
RMSD/RMSF and intra- and intermolecular residue-residue contact maps. (**a**), (**b**) RMSD/RMSF of the Cα -atoms of WT-TIMP1 (blue) and p.L245P-TIMP1 (red) residues during three independent 1 μs MD simulations with Mdn (Range). (**c**) Average intra- and intermolecular residue-residue contact maps: spectrum range from blue (100%) to white (0% ) to red (− 100%); blue = more contacts in WT, red = more contacts in p.L245P; each point represents the mean of the average residue-residue contacts (last 200 ns of MD simulation time); center of mass < 5 Å = contact; MMP10: specificity loop (Tyr236-Ser251); TIMP1: multiple turn loop of C-terminal subdomain (Gln150-Lys157).

**Figure 4:**
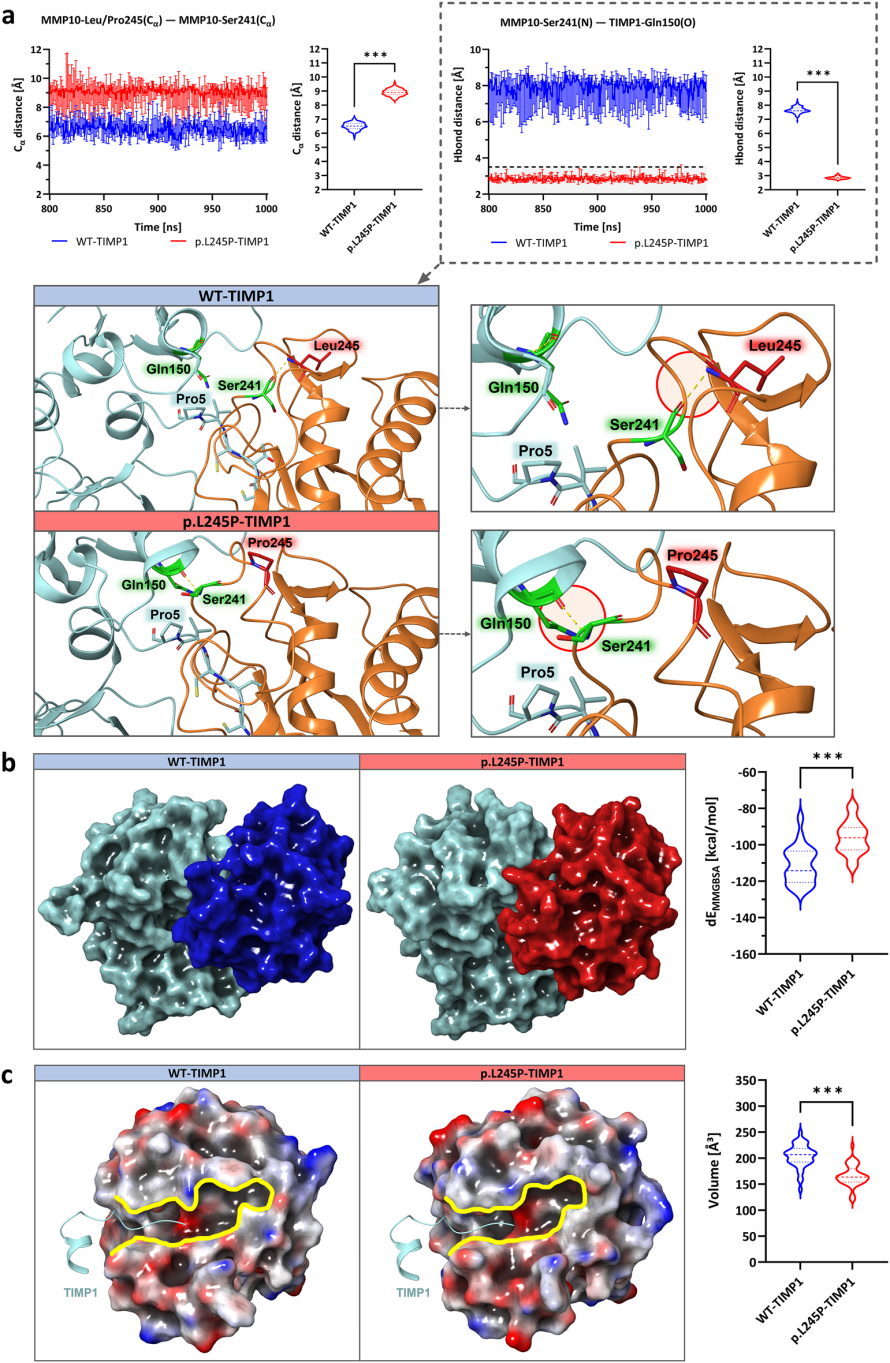
Analysis of residue-residue distances, MM/GBSA, and volume of the substrate-binding cleft of WT/p.L245P-TIMP1. (**a**) Cα- and H-bond distance analyses of WT/p.L245P-TIMP1 residues: MMP10 (ribbon, turquoise), TIMP1 (ribbon, red), Pro5, Gln150, Ser241, and Leu245/Pro245 (stick), major H-bond distance differences (circled). (**b**) Surface of WT-TIMP1 (MMP10: turquoise; TIMP1: blue) and p.L245P-TIMP1 (MMP10: turquoise; TIMP1: red) and the total free binding energy analysis of WT/p.L245P-TIMP1. (**c**) Surface of the WT/p.L245P-TIMP1 (colored by electrostatic potential from red to white to blue) with substrate-binding cleft (yellow) and volume substrate-binding cleft. All data: WT-TIMP1 compared to p.L245P-TIMP1 (last 200 ns of MD simulation time), U-test, violin plots, Mdn (range), n.s. = not significant, * = *p* < 0.05, ** = *p* < 0.01, *** = *p* < 0.001.

MD simulations were performed to investigate the dynamic characteristics of MMP10-TIMP1 binding behavior upon insertion of the p.L245P variant, as previously studied by our research group for other gene mutations^26–28^. Figure 2A shows a schematic display of MMP10-TIMP1 with the major protein segments highlighted, such as the affected amino acid Leu245 in the specificity loop; the αB helix with the characteristic zinc-binding motif *HEXXHXXGXXH*, which binds the catalytic zinc via His217, Glu218, His221, His227, and is responsible for the proteolytic activity; the methionine-turn with Met235, which provides a hydrophobic base for the zinc-binding site; and the N-terminal TIMP1 segment Cys1-Pro5, which binds to the substrate-binding cleft of MMP10. RMSD analyses (Fig. 3a) show that both models reached an equilibrium state within a simulation time of 1 µs (WT-TIMP1: Mdn = 2.7 Å (IQR = 0.1); p.L245P-TIMP1: Mdn = 2.4 Å (IQR = 0.1). The different conformational states sampled for both models showed no major differences (Supplementary Fig. S1). In addition, RMSF analyses were comparable over the entire length of both models [WT-TIMP1: Mdn = 0.8 Å (IQR = 0.4); p.L245P-TIMP1: Mdn = 0.8 Å (IQR = 0.5)]. The only significant RMSF differences (Fig. 3b) were observed in the Ser241-Leu245 region in the specificity loop of MMP10 by 0.6 Å [WT-TIMP1: Mdn = 1.1 Å (IQR = 0.5); p.L245P-TIMP1: Mdn = 1.7 Å (IQR = 0.2); *p* = 0.02] and in the entire multiple turn loop of C-terminal subdomain of TIMP1 (Gln150-Lys157) by 0.7 Å [WT-TIMP1: Mdn = 0.9 Å (IQR = 0.2); p.L245P-TIMP1: Mdn = 1.7 Å (IQR = 0.7); *p* < 0.001]. Superimposition of WT/p.L245P-TIMP1 structures (every 10th snapshot of the last 200 ns of MD simulation time) showed a different arrangement of loops. For example, the flexible amino acid Leu245 in WT-TIMP1 appeared to exert a larger range of motion than the rigid amino acid Pro245 in p.L245P-TIMP1. In addition, Cys1-Pro5 of TIMP1 appeared to have a different structural alignment in the substrate-binding cleft of MMP10 in p.L245P-TIMP1 (Fig. 2B). Average intra- and intermolecular residual contact maps were created to assess the differences in the interactions between WT/p.L245P-TIMP1 structures. The largest and most frequent intra- and intermolecular residue-residue contact differences were found in the specificity loop of MMP10 and the multiple turn loop of the C-terminal subdomain of TIMP1 (Fig. 3c). In the specificity loop of MMP10, there are 10 different intramolecular contacts each, present ≥ 30% of the time only in WT models and only in p.L245P models. Between the specificity loop (MMP10) and the multiple turn loop of the C-terminal subdomain (TIMP1), there were 13 different intermolecular contacts in WT-TIMP1 models and 16 different intermolecular contacts in p.L245P-TIMP1 models, present ≥ 30% of the time. Figure 4a shows that particularly in the Leu245/Pro245 region, there is a larger intramolecular Cα-atom distance between Pro245-Ser241 in p.L245P-TIMP1 [Mdn = 8.9 Å (IQR = 0.3)] compared to Leu245-Ser241 in WT-TIMP1 [Mdn = 6.5 Å (IQR = 0.4)] (*p* < 0.001). Therefore, Ser241(N) is probably unable to exert intermolecular H-bonding to TIMP1-Gln150(O) in WT-TIMP1 [Mdn = 7.6 Å (IQR = 0.3)], whereas Ser241(N) exerts H-bonding to TIMP1-Gln150(O) in p.L245P-TIMP1 [Mdn = 2.8 Å (IQR = 0.1)] due to its shorter distance (*p* < 0.001). To determine whether individual residue-residue contact changes could cause a difference in the overall ligand-binding affinity of WT/p.L245P-TIMP1 structures, the total free binding energy, dEMMGBSA [kcal/mol], was calculated (Fig. 4b). The data showed that the p.L245P variant led to a 16% weaker binding affinity between p.L245P-TIMP1 structures [Mdn = − 96.2 kcal/mol (IQR = 12.4)] compared to WT-TIMP1 structures [Mdn = − 114.0 kcal/mol (IQR = 18.0)] (*p* < 0.001). The results of volume measurements (Fig. 4c) showed that p.L245P-TIMP1 structures [Mdn = 163.6 Å^3^ (IQR = 25.8)] have a 21% lower volume of the substrate-binding cleft compared to WT-TIMP1 structures [Mdn = 206.5 Å^3^ (IQR = 26.1)] (*p* < 0.001).

### THP-1 cell transfection, overexpression, and knockdown

**Figure 5:**
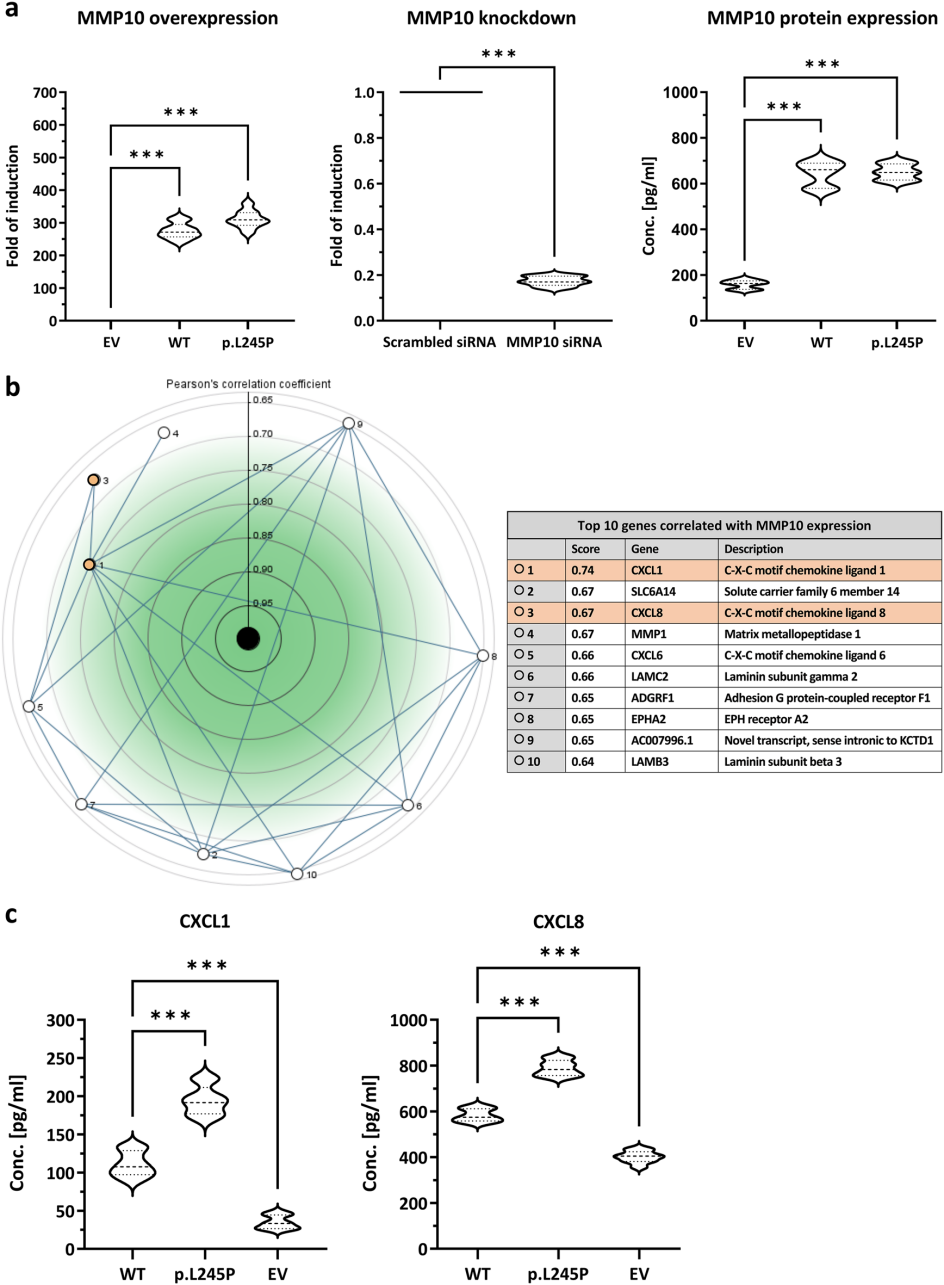
MMP10 overexpression, knockdown, protein expression, gene Co-expression, and CXCL1/CXCL8 expression. (**a**) Left: *MMP10* overexpression with quantitative RT-qPCR, expressed as fold-change induction. Middle: *MMP10* knockdown with siRNA expressed as fold-change. Right: Quantification of MMP10 protein expression, indicating its concentration; EV = empty vector, (**b**) Gene co-expression of *MMP10* (center) with a description of the top 10 most correlated genes (correlation > 0.643 and mutual correlation ≥ 0.601). (**c**) Quantitative determination of CXCL1 and CXCL8 concentrations using ELISA; All data: n = 9, U-test, violin plots, Mdn (IQR), n.s. = not significant, * = *p* < 0.05, ** = *p* < 0.01, *** = *p* < 0.001.

Reliable and efficient transfection of THP-1 cells is important for experimental validity. Electroporation has previously been described as a successful method for the transfection of THP-1 cells for plasmid DNA overexpression because it does not affect macrophage differentiation and polarization while maintaining cell viability^29–31^. Using electroporation, we achieved a mean transfection efficiency of 86% when measuring the GFP ctrl plasmid (Supplementary Fig. S2) and a *MMP10* gene induction (Fig. 5a) in WT [Mdn = 271.2 (IQR = 38.2)] and p.L245P [Mdn = 309.1 (IQR = 38.4)] (*n* = 9, *p* < 0.001)*.* Using Lipofectamine 2000, siRNA-mediated knockdown efficiency (Fig. 5a) was achieved with a reduction in *MMP10* gene expression to 14–20% of the control level [Mdn = 0.17 (IQR = 0.04)] (*n* = 9*, p* < 0.001)*.* Quantification of MMP10 expression in cell culture medium by ELISA (Fig. 5a) showed increased protein concentration in WT [Mdn = 660.6 pg/mL (IQR = 109.9)] and p.L245P [Mdn = 648.5 pg/mL (IQR = 70.4)] (*n* = 9*, p* < 0.001)*.* In conclusion, the overexpression of MMP10 with the p.L245P and WT variant at the gene and protein level, and its knockdown with siRNA was successfully achieved.

### Impact of p.L245P variant on CXCL1 and CXCL8 expression

We first used the Genvestigator evaluation of gene co-expression with *MMP10* from Affymetrix Human Genome U133 Plus 2.0 data and showed that CXCL1 (correlation score = 0.74) and CXCL8 (correlation score = 0.67) were among the three genes that correlated most strongly with *MMP10* (Fig. 5b). Therefore, we aimed to validate these findings and assessed the impact of the p.L245P variant on the secretion of CXCL1 and CXCL8. Figure 5c illustrates that a quantitative determination of CXCL1 levels by ELISA in p.L245P-macrophages [Mdn = 191.6 pg/mL (IQR = 34.4)] showed a 78% higher expression compared to WT-macrophages [Mdn = 107.7 pg/mL (IQR = 31.6)] (*p* < 0.001) and a 5.8-fold higher expression compared to control empty-vector (EV) macrophages [Mdn = 33.3 pg/mL (IQR = 18.0)] (*n* = *9, p* < 0.001)*.* A quantitative determination of CXCL8 concentration by ELISA revealed a similar distribution pattern with 36% higher expression in p.L245P-macrophages [Mdn = 783.3 pg/mL (IQR = 66.0)] compared to WT-macrophages [Mdn = 574.1 pg/mL (IQR = 54.0)] (*p* < 0.001) and a 1.9-fold higher expression compared to control EV-macrophages [Mdn = 404.4 pg/mL (IQR = 42.5)] (*n* = *9, p* < 0.001)*.* In conclusion, our data showed higher expression of CXCL1 and CXCL8 in p.L245P-macrophages than in WT and EV-macrophages.

### Impact of p.L245P variant on macrophage adhesion and migration

**Figure 6:**
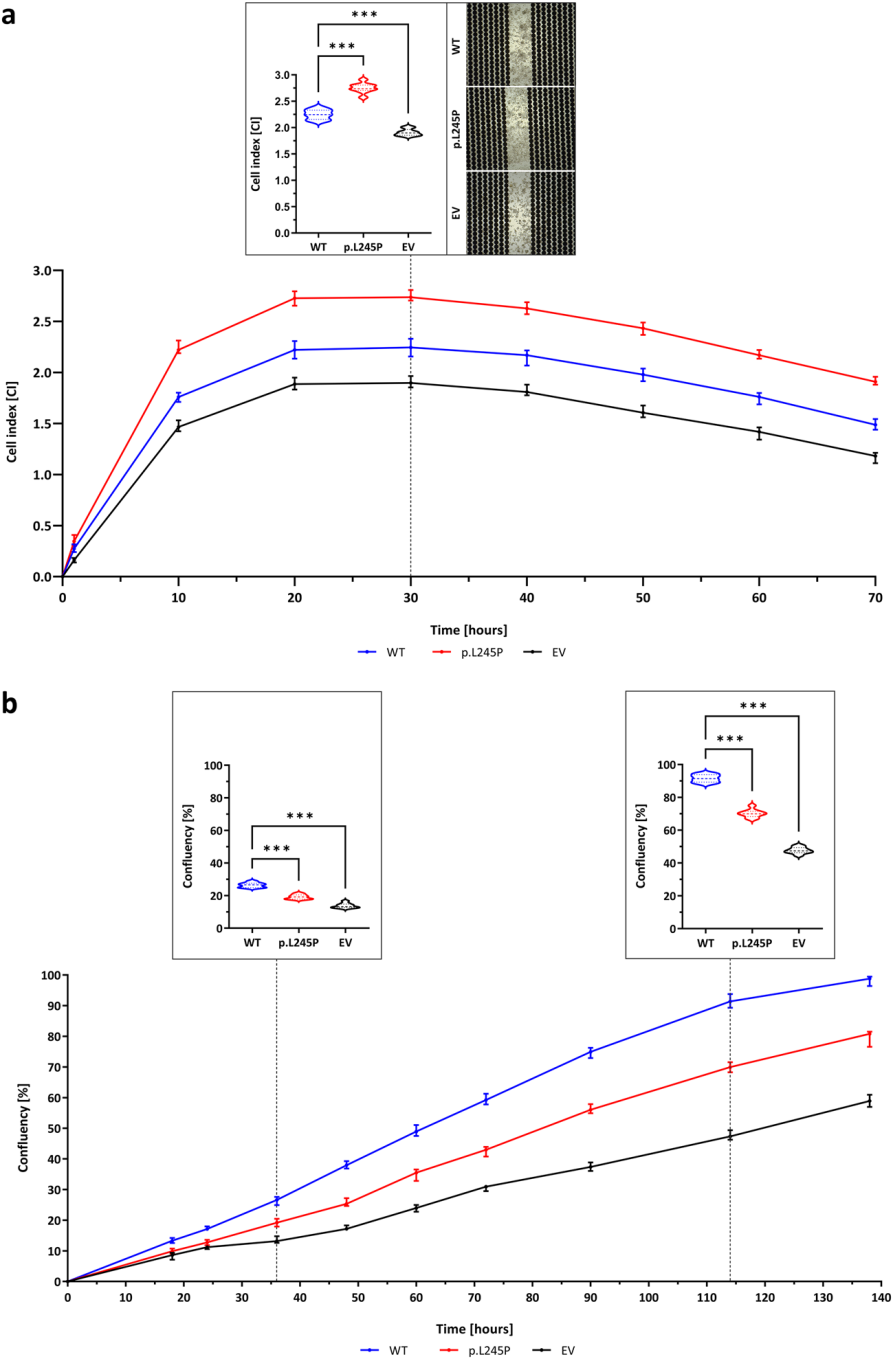
Macrophage adhesion and migration, Gene Co-expression of *MMP10*, CXCL1 and CXCL8 expression. (**a**) Impedance-based real-time analysis of macrophage adhesion over a 72-h period with calculated cell index. (**b**) Macrophage migration analysis over a 138-h period with calculated cell confluence. All data: n = 9, U-test, violin plots, Mdn (IQR), n.s. = not significant, * = *p* < 0.05, ** = *p* < 0.01, *** = *p* < 0.001.

An adhesion and migration assay was performed to investigate whether p.L245P exhibits different adhesion and migration behaviors than WT as processes regulating atherosclerotic progression. The evaluation of the real-time impedance-based analysis on gold microelectrode E-plates showed different cell adhesion behaviors with diverse cell index (CI) values between WT, p.L245P, and EV throughout the 72-h observation period (Fig. 6a). In the first stage of adhesion, a rapid increase in CI value was observed for all samples, resulting in a 26% higher CI value for p.L245P [Mdn = 2.2 (IQR = 0.1)] than for WT [Mdn = 1.8 (IQR = 0.1)] after 10 h (*p* < 0.001). In the second stage of adhesion, there was a continuous increase in CI values for all samples until confluence of the golden microelectrodes was reached after 20 h. The subsequent plateau stage of adhesion ended at 30 h, resulting in a 22% higher CI value for p.L245P [Mdn = 2.7 (IQR = 0.1)] than for WT [Mdn = 2.3 (IQR = 0.2)] (*n* = *9, p* < 0.001)*.* After 30 h, the macrophages detached from the microelectrodes, leading to a continuous decrease in the CI value. Impedance-based analysis of macrophages in the 72-h period shows that overexpression of MMP10 resulted in higher CI values and p.L245P-macrophages had increased adhesiveness to microelectrodes compared to WT-macrophages.

The results of the migration assay with measurements of cell confluence during the 138 h in culture inserts showed a different cell migration behavior of WT-/p.L245P-/EV-macrophages (Fig. 6b). For example, after 36 h, 39% higher cell confluence was measured for WT-macrophages [Mdn = 26.6% (IQR = 2.7)] than for p.L245P-macrophages [Mdn = 19.2% (IQR = 2.6)] (*p* < 0.001). After 114 h, WT-macrophages [Mdn = 91.4% (IQR = 4.4)] had 1.3-fold higher cell confluence than p.L245P-macrophages [Mdn = 69.9% (IQR = 3.3)] (*p* < 0.001) and 1.9-fold higher cell confluence than control EV-macrophages [Mdn = 47.4% (IQR = 3.1)] (*n* = 9*, p* < 0.001)*.* In summary, the calculation of cell confluence at 138 h shows that p.L245P-macrophages have reduced cell confluence and cell-front migration rates compared to WT-macrophages.

## Discussion

Using whole-exome sequencing (WES) and analyzing the exome data of 255 unrelated patients with MI, we identified the p.L245P variant in the *MMP10* gene. The p.L245P variant is located in the specificity loop (Tyr236-Ser251) of MMP10, which is important for substrate specificity and is the region with the greatest variation among all MMPs^22^. In MD simulations, the p.L245P variant changed the intra- and intermolecular residue-residue contacts of MMP10-TIMP1, particularly between the specificity loop of MMP10 and the multiple turn loop of the C-terminal-subdomain (Gln150-Lys157) of TIMP1. This presumably resulted in a different H-bonding network with a weaker binding affinity between the p.L245P-TIMP1 structures and the volume-minimized substrate-binding cleft of MMP10 (Figs. 3c and 4b/c). This results in an altered protein-protein interaction that could reduce MMP10 inhibition^32^. The analyses of this study were limited to TIMP1 because it is widely distributed in human tissues, particularly in regions of inflammation^33,34^. An imbalance of MMP-TIMP1 has been described in other severe diseases^33^, thus therapeutically selective MMP inhibitors (MMPIs) have been the subject of research for over 50 years. The challenge in developing MMPIs is to achieve specificity^35^. Therefore, the identification of *MMP10* variants with detailed atomic structures of specific pockets in the substrate-binding cleft is essential for the development of selective MMP10 inhibitors.

MMP10 is known to be involved in macrophage adhesion, migration, and invasion. It was shown that RNAi silencing of *MMP10* in primary macrophages reduced the migration ability of the cells, and exogenous active MMP10 protein was able to reverse this effect^15^. Not only endothelial adhesion and migration of circulating monocytes by focal adhesion molecules are relevant for the pathogenesis of atherosclerosis, but also transendothelial and mesenchymal migration of macrophages to sites of inflammation by podosomes^36,37^. The results of our in-vitro assays showed that the p.L245P variant in the *MMP10* gene resulted in increased adhesion and decreased migration ability of macrophages compared to WT-macrophages. Increased accumulation of M1-macrophages due to increased macrophage adhesion is known to promote the progression of atherosclerotic plaques^38^. Additionally, increased macrophage adhesion leads to decreased macrophage migration in the ECM^39^, resulting in reduced plaque macrophage migration^40^, greater proliferation of lesional macrophages, and enhanced retention of VSMCs and lipoproteins^41^. Possible reasons for the reduced and delayed migration of p.L245P-macrophages could be the previously mentioned increased cell adhesion ability of p.L245P-macrophages, or a slower formation of lamellipodia with a different distribution pattern of podosomes at the cell front^42,43^.

Macrophages with increased expression of pro-inflammatory chemokines contribute to an enhanced inflammatory response^44^. CXCL1 and CXCL8 were among the top three most correlated *MMP10* genes (Fig. 5b). CXCL1 and CXCL8 recruit neutrophils to sites of inflammation via the common proatherogenic receptor CXCR2^45^. Previous studies have shown that higher CXCL1 expression is present in patients with unstable angina, enhances the secretion of MMPs^46^, and activates macrophages in atherosclerotic lesions^47^. CXCL8, on the other hand, promotes the pro-inflammatory activity of macrophages^48^, has angiogenic properties^49^, and leads to inhibition of TIMP1 expression in macrophages with increased MMP activity in atherosclerotic plaques^50,51^. In our study, p.L245P-macrophages expressed higher levels of CXCL1 and CXCL8 than WT-macrophages and thus may exert a pro-inflammatory effect in the pathogenesis of atherosclerosis.

In conclusion, the findings of our in-silico and in-vitro studies provide evidence that the p.L245P variant in *MMP10* may contribute to a pro-atherosclerotic effect, promoting plaque destabilization and rupture in affected family members. This effect is likely mediated by altered MMP10-TIMP1 interactions, increased macrophage adhesion, reduced macrophage migration, and elevated expression of CXCL1 and CXCL8 by macrophages.

In our study, the children of the affected parents are currently younger than the age of disease manifestation in their parents at 37–49 years and have not suffered from MI to date. Data from the prospective population-based Framingham Study since 1948 showed that a parental history of death due to CAD is an independent cardiovascular risk factor for the premature occurrence of the disease^52^. A follow-up study of the 1971 offspring cohort showed that cardiovascular disease in parents was associated with a threefold higher event rate in offspring aged 30–59 years^53^. Therefore, the development and promotion of specific prevention strategies are of great importance because of the increased risk of atherosclerosis in the middle-aged offspring of affected families.

Nevertheless, it is important to recognize the limitations of our study: the use of THP-1 cells instead of primary cells and a moderate number of experimental replicates for in-vitro studies (n = 9). We recommend further investigations using monocytes from donors carrying the p.L245P variant to substantiate our hypothesis. Also studying the effect of this mutation in atherogenic animal models will be of great importance. Furthermore, the small sample size of the two families precludes definitive conclusions about the co-segregation of p.L245P and the MI phenotype. Despite this constraint, the notable discrepancy in allele frequency—0.001 in the European (non-Finnish) population, as reported by the Genome Aggregation Database^54^, versus 0.008 in our cohort of 255 MI patients—strongly supports that this variant is indeed the causal genetic risk factor for MI in our families.

## Materials and methods

### Variant identification

WES of 255 patients with premature MI from the population-based German-MI-Family-Study (GerMIFS) was performed by the Institute of Human Genetics at the Helmholtz Center Munich to identify rare disease-causing variants^55^. Here, we screened the data for variants in *MMP10* and identified six variants (Supplementary Table S1). The variant NM_002425:exon5:c.T734C:p.L245P was found in two unrelated families and co-segregated with the disease in the respective families and was chosen for further analysis in this study. Apart from chr11:102650461A > T (which was used for analysis in another study), all other variants did not show co-segregation and were therefore not examined in more detail. The study was approved by the local Ethics Committee of the University of Regensburg (Germany) and all methods were performed in accordance with the relevant guidelines and regulations of the respective institution. All participating individuals gave informed consent.

### Molecular dynamics simulations

The X-ray crystal structure of MMP10-TIMP1 was selected from the Protein Data Bank (PDB 10.2210/pdb3V96/pdb), and the p.L245P variant was inserted for comparison. Both structures were prepared using the Protein Preparation Wizard (SCHRÖDINGER®2020–3)^56,57^, including the assignment of force field atom types and bond orders, addition of hydrogen atoms, termini capping, calculation of incomplete side chains and loops, generation of heterogeneous states at pH 7 ± 2, and optimization of hydrogen bond (H-bond) networks by minimization of sampled hydrogens. Minimization was performed using the OPLS3e force field with a convergence threshold of 0.05^58^. The resulting structures were subjected to solvent-explicit MD simulations for all atoms using the GPU-accelerated SCHRÖDINGER®DESMOND software^59,60^. Both structures were integrated into a minimized periodic simple point-charged water model with an orthorhombic box size of 10.0 Å × 10.0 Å × 10.0 Å and 0.15 M NaCl ions. The MD simulations were run with periodic boundary conditions in the NPT ensemble (T = 300 K and P = 1 bar) using OPLS3e force field parameters. Nose–Hoover temperature coupling and isotropic scaling were used to control temperature and pressure. Finally, a 1  µs NPT production simulation was carried out for each structure with storage at 1 ns intervals, in threefold independent repetition. Simulations were then compared using root mean square deviation (RMSD) and root mean square fluctuation (RMSF). For all analyses, the course of the last 200 ns of the simulation time (every 10th snapshot) and the mean calculated from triplicate measurements were used. The intra- and intermolecular residue-residue contacts of WT/p.L245P-TIMP1 with a distance of < 5 Å between the centers of mass were monitored using the SCHRÖDINGER® trajectory_asl_monitor.py script. The interaction energy of MMP10-TIMP1 was determined for both structures using the Molecular Mechanics (MM)/Generalized Born Surface Area (GBSA) method^61^ with the thermal_mmgbsa.py script from SCHRÖDINGER®. The SCHRÖDINGER® trajectory_binding_site_volumes.py script was used to calculate the volume of the substrate-binding cleft in both structures. Illustrations were made using SCHRÖDINGER® Maestro 2020-3^62^.

### Plasmid constructs

The *MMP10* (NM_002425) Human Tagged ORF Clone (OriGene, Cat. RC200453) was used as a plasmid cDNA for the insertion of the p.L245P variant (c.734 T > C) and to create an empty vector (EV) as a control by the Institute of Cardiogenetics at the University of Luebeck using site-directed mutagenesis. An expression clone was generated in collaboration with the German Heart Center in Munich using pCMV6-Entry (OriGene, Cat. PS100001) as the entry vector and Gateway™ pcDNA™-DEST40 (Invitrogen, Cat. 12274015) as the destination vector.

### Cell culture and transfection of THP-1 cells

Human THP-1 monocyte-like cells (Cytion, Cat. 300356) were cultured and expanded. Subcultivation with 1 × 10^5^ cells/mL was performed every 48 h (4-week period) in a culture medium consisting of 450 mL of RPMI-Medium 1640 GlutaMAX (Gibco, Cat. 61870036), 50 mL of FKS Gold Plus (Bio & Sell, EAN. FBSGP0500), and 5 mL of MEM NEAA 100x (Gibco, Cat. 11140050). The chosen transfection method for *MMP10* overexpression was electroporation by Nucleofector technology using Amaxa Nucleofector II (Lonza, Cat. AAB-1001). Stock solutions of each plasmid DNA (WT, p.L245P, and EV) were diluted to 0.5 µg as the maximum recommended DNA amount per sample for THP-1 cells. The Amaxa Cell Line Nucleofector Kit V (Cat. VCA-1003) with the optimized protocol for THP-1 cells was used for transfection, with green fluorescent protein (GFP) as a control. Cell staining was performed with DAPI (Sigma-Aldrich, CAS No. 28718903). Cell imaging was performed using Keyence BZ-9000 microscope. For knockdown experiments, Lipofectamine 2000 reagent (Invitrogen, Cat. 11668019) was used for siRNA transfection according to the manufacturer's protocol, including BLOCK-iT Alexa Fluor Red Fluorescent (Invitrogen, Cat. 14750100) as a control. Each transfection sample per well (24-well plate) was composed of 500 µL of PMA-stimulated cell suspension with 4 × 10^5^ cells/mL, 1.5 µL of Lipofectamine 2000, and 100 µL of Opti-MEM reduced serum medium (Gibco, Cat. 31985062), and 3 µL of siRNA with the target sequence *5*′*AACAAGGATCTTGCCCAGCAA3*′ (1 nmol; Qiagen, FlexiTube GeneSolution, Cat. 1027416). 3 µL of AllStars Neg. siRNA (20 nmol; Qiagen, Cat. 1027281) and a scrambled siRNA with the target sequence *5*′*CGAACUCACUGGUCUGACCTT3*′ (20 nmol; IBA-Lifesciences) were used as negative controls. After 24 h of PMA stimulation and siRNA transfection, THP-1 cells were harvested and prepared for RNA isolation.

### THP-1 cell differentiation

After transfection, THP-1 cells (4 × 10^5^/mL) were differentiated for 48 h using phorbol-12-myristate-13-acetate (PMA; Sigma-Aldrich, Cat. P8139) in a 12-well plate containing 3 mL of medium and 3 µL PMA (concentration of 100 ng/µL) per well. The cell culture medium was changed after 48 h of incubation. After a further 24 h of cell recovery, the medium supernatant was collected and centrifuged, and adherent macrophages were detached with 1 mL Accutase Solution (Sigma-Aldrich, Cat. A6964) per well for a maximum of 30 min.

### RNA isolation and RT-qPCR

RNA was isolated using a RNeasy Plus Mini Kit (Qiagen, Cat. 74136) according to the manufacturer's protocol. The RNA samples were diluted to 100 ng/µL for reverse transcription. cDNA synthesis from 1 µg of RNA was performed using M-MLV Reverse Transcriptase (200 U/µL; Invitrogen, Cat. 28025021). *MMP10* gene expression was determined by RT-qPCR in a 7900HT Fast Real-Time PCR System using PowerUP SYBR Green Master Mix (Applied Biosystems, Cat. A25742), and *F: 5′-CACAGTTGGCTCATGCCTA-3′*; *R: 5′-GCTTCAGTGTTGGCTGAGTG-3′* as primers for *MMP10*. Arithmetic means of multiple replicates were used to determine relative gene expression. The threshold cycle numbers of *GAPDH* and *MMP10* were used to determine the fold changes in expression as 2^-ddCt^.

### MMP10 ELISA

MMP10 expression in cell culture medium was quantified using the MMP10 Human ELISA Kit (Invitrogen, Cat. EHMMP10) with a detection range of 2.06-1500 pg/mL according to the manufacturer's protocol.

### THP-1 cell adhesion

Impedance-based real-time analysis was performed using an xCELLigence Real-Time Cell Analyzer (ACEA-Bio, Cat. 00380601030) for 72 h to investigate macrophage adhesion^63^. Changes in the electrode impedance of E-Plate 16 gold microelectrode sensors (ACEA-Bio, Cat. 05469830001) are expressed by the dimensionless CI. A concentration of 4 × 10^4^ cells/mL per well was determined. The assay was performed according to the manufacturer's instructions.

### THP-1 cell migration

For macrophage migration, culture inserts for self-insertion (4 wells, Ibidi, Cat. 80469) were used with a cell concentration of 1.5 × 10^6^/mL and a maximum filling volume of 110 µL per well. After 48 h, the culture inserts were removed, and bright-field images of the cell-free gaps were obtained for 138 h with the Olympus IX70 using a 10 × phase-contrast objective. The image acquisitions were implemented in Python 3.6 (PyCharm-JetBrains) and the cell confluency was calculated using an in-house Python script. The cell culture medium was changed every 12 h to remove detached cells.

### CXCL1 and CXCL8 expression

To identify the inflammatory genes most correlated with *MMP10*, co-expression analysis from Affymetrix Human Genome U133 Plus 2.0 data (254 anatomical parts) was performed using the Genvestigator gene expression database. The top ten most correlated genes with a correlation above 0.643 were filtered (https://genevestigator.com/-access 26.09.2022). Subsequently, CXCL1 and CXCL8 were selected as candidates for quantitative determination using ELISA. The Human CXCL1 ELISA Kit (Invitrogen, Cat. EHCXCL1) with a detection range of 1.37-1000 pg/mL and the Human IL-8 ELISA Kit (Invitrogen, Cat. KHC0081) with a detection range of 15.6-1000 pg/mL were performed according to the manufacturer's protocol.

### Statistical analysis

All tests were performed with three samples each in threefold independent replicates, resulting in a sample number of n = 9 per data group. The nonparametric Mann-Whitney-Wilcoxon test (U-test) was performed to compare the ranks (no Gaussian distribution), calculating a two-tailed *p*-value with a confidence interval of 95% and a significance level of *α* = 0.05. Violin plots, medians (Mdn), and interquartile ranges (IQR) were applied to the descriptive statistics of the graphs. GraphPad Prism 9.5.1 (GraphPad Software, Inc.) was used for the statistical analysis.

### Supplementary Information

Supplementary Legends.

Supplementary Figure S1.

Supplementary Figure S2.

Supplementary Legends.

Supplementary Table S1.

## Data Availability

The data sets generated and analyzed in the current study are available in the gnomAD v2.1.1 database (SNV:11-102647396-A-G(GRCh37)), the dbSNP database (rs147985027), the ClinGen Allele Registry (CA6249990), the UCSC Genome Browser on Human (GRCh37/hg19), and in the RCSB PDB (3V96). gnomAD v2.1.1 database (SNV:11-102647396-A-G(GRCh37)) https://gnomad.broadinstitute.org/variant/11-102647396-A-G?dataset=gnomad_r2_1. dbSNP database (rs147985027) https://www.ncbi.nlm.nih.gov/snp/rs147985027. ClinGen Allele Registry (CA6249990) https://reg.clinicalgenome.org/redmine/projects/registry/genboree_registry/by_canonicalid?canonicalid=CA6249990. UCSC Genome Browser on Human (GRCh37/hg19) https://genome.ucsc.edu/cgi-bin/hgTracks?db=hg19&lastVirtModeType=default&lastVirtModeExtraState=&virtModeType=default&virtMode=0&nonVirtPosition=&position=chr11%3A102647371%2D102647421&hgsid=2244763264_vIse85X6cfaCoRor5IP5VIJDtfFW. RCSB PDB (3V96) 10.2210/pdb3V96/pdb.
