# Supplementary material for: Identification of a functional missense variant in the matrix metallopeptidase 10 (MMP10) gene in two families with premature myocardial infarction
Source: Sci Rep. 2024 May 28;14:12212. doi: 10.1038/s41598-024-62878-3 (PMC11133425; doi:10.1038/s41598-024-62878-3)
Supplement: Supplementary file 1 — Supplementary Legends. [file 41598_2024_62878_MOESM1_ESM.docx]

Supplementary Fig. S1. Frequency Distribution of RMSD Cα [Å] calculations for WT-TIMP1 and p.L245P-TIMP1.

Representation of the different conformational states for both WT-TIMP1 and p.L245P-TIMP1, in triplicate, showing the relative frequency of RMSD Cα calculations (WT-TIMP1_01: dark blue, WT-TIMP1_02: blue; WT-TIMP1_02: light blue; p.L245P-TIMP1_01: burgundy; p.L245P-TIMP1_02: red; p.L245P-TIMP1_03: orange).

Supplementary Fig. S2. THP-1 cell transfection, DAPI cell staining, and transfection efficiency.

(a) Left: THP-1 cells were transfected with green fluorescent protein (GFP) as a control for *MMP10* overexpression using electroporation. Right: THP-1 cells were transfected with BLOCK-iT Alexa Fluor Red Fluorescent as a control for siRNA transfection using Lipofectamine 2000. Cell imaging was performed with 4x and 10x objectives using a Keyence BZ-9000 microscope. (b) Images of three independent transfections at 10x objective showing transfection efficiency after counting DAPI-stained nuclei (blue) and GFP-stained cells (green) after transfection by electroporation.
