# Supplementary figures and images for: Identification of a functional missense variant in the matrix metallopeptidase 10 (MMP10) gene in two families with premature myocardial infarction
Source: Sci Rep. 2024 May 28;14:12212. doi: 10.1038/s41598-024-62878-3 (PMC11133425; doi:10.1038/s41598-024-62878-3)

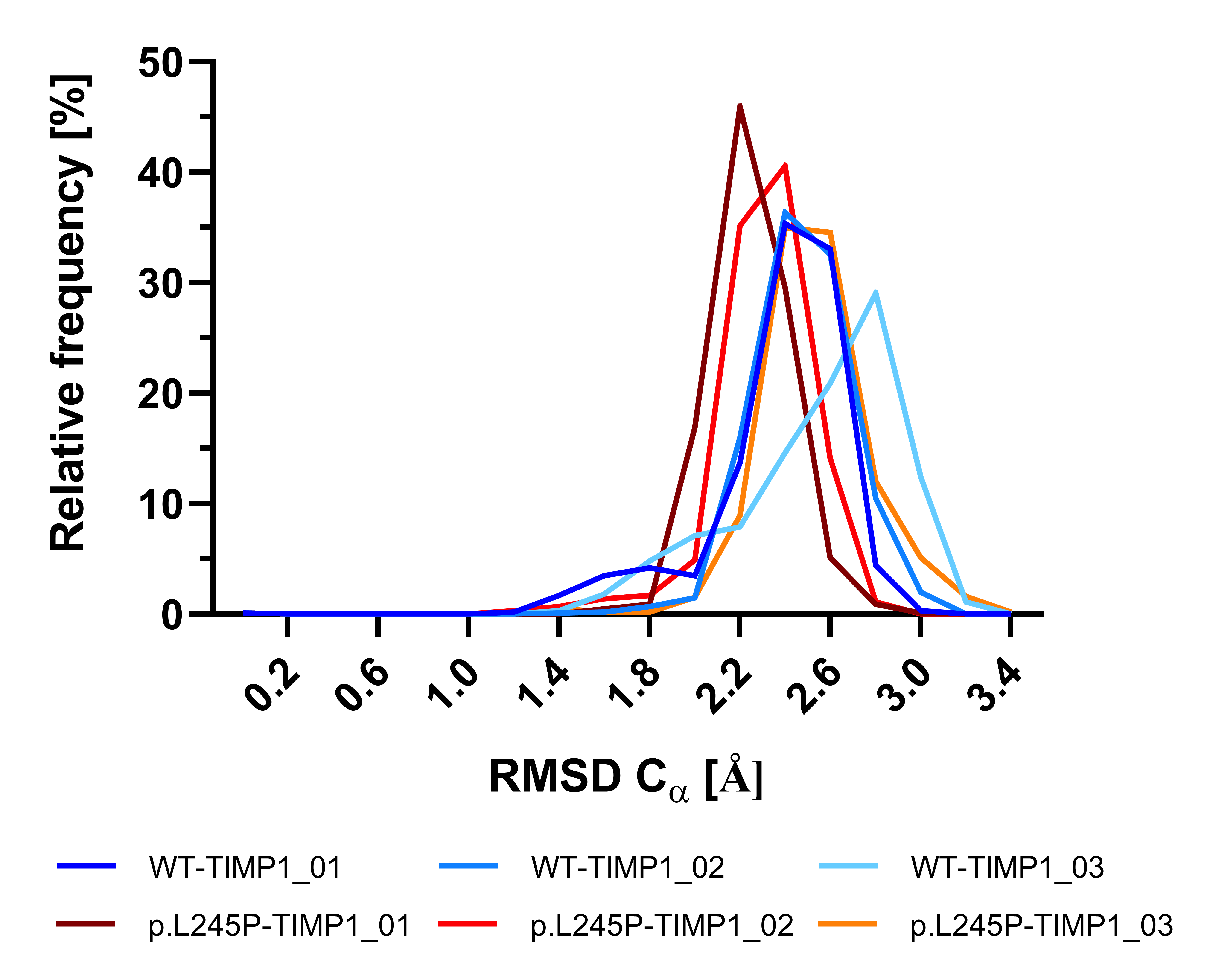

Supplement: Supplementary file 2 — Supplementary Figure S1. [file 41598_2024_62878_MOESM2_ESM.tiff]

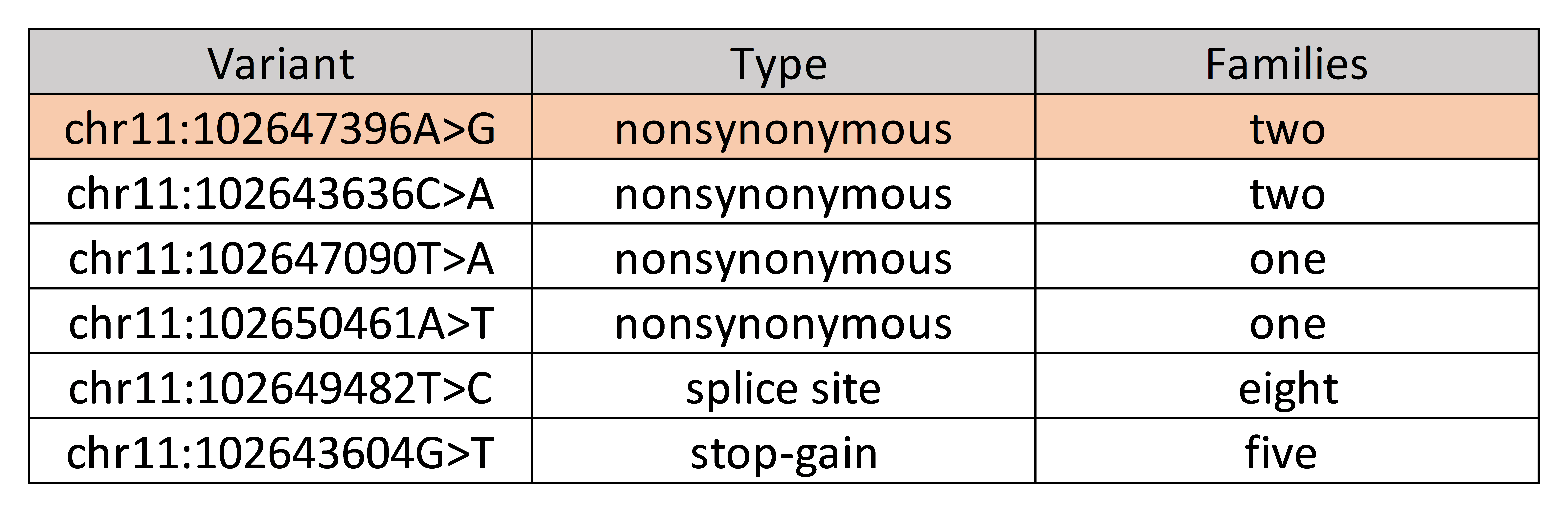

Supplement: Supplementary file 5 — Supplementary Table S1. [file 41598_2024_62878_MOESM5_ESM.tif]
