# Supplementary material for: Identification of a functional missense variant in the matrix metallopeptidase 10 (MMP10) gene in two families with premature myocardial infarction
Source: Sci Rep. 2024 May 28;14:12212. doi: 10.1038/s41598-024-62878-3 (PMC11133425; doi:10.1038/s41598-024-62878-3)
Supplement: Supplementary file 4 — Supplementary Legends. [file 41598_2024_62878_MOESM4_ESM.docx]

Supplementary Table S1. Identification of six MMP10 variants in 255 MI patients.

Representation of the six MMP10 variants after data screening of 255 MI patients, indicating variant type and number of affected families. Highlighted in orange: investigated variant of this study (NM_002425:exon5:c.T734C:p.L245P).
